# News and Notes

**Published:** 1900-09

**Authors:** 


### *NEWS AND NOTES.*

Dr. F. Savary Pearce has been elected clinical professor of diseases of the nervous system in the Medico-Chirurgical College of Philadelphia, succeeding Dr. C. W. Burr, resigned.

Dr. Joseph Leidy, of Philadelphia, was appointed a member of the Jury on Hygiene from the United States at the International Congress on Hygiene, which met in Paris August 4.

Dr. Edwin Klebs, who for a number of years has been a resident of the United States and for the last two professor of bacteriology in Rush Medical College, Chicago, has resigned this latter position and will return to Germany where in the future he will reside.

Philadelphia Hospital.—Dr. E. S. Gans has been appointed on the staff to succeed Dr. J. Abbott Cantrell, resigned. Dr. M. B. Hartzell was also elected a member of the corps. Dr. Ella M. Anderson was elected assistant physician to the department for the insane.

The Roentgen Society.—The Roentgen Society of the United States will meet in New York City, December 13 and 14, 1900, at the Academy of Medicine. Papers have been promised by eminent men abroad and here, and a very successful scientific meeting is assured.

In the opinion of a Berlin physician, Dr. Rosenbach, the causes of that annoying and persistent redness of the tip of the nose, which is particularly frequent among women of a delicate complexion, are the pressure of the veil and the friction produced by it. Treatment consists primarily in the disuse of the veil.

---

NOTICE.—All surgeons, assistant surgeons, acting assistant surgeons or contract surgeons, and hospital stewards, who served in the army or navy of the late Confederate States, will please send their post-office address to Deering J. Roberts, M.D., Secretary Surgeons' Association, C. S. A., Nashville, Tenn.

---

PENNSYLVANIA HOSPITAL.—Dr. Robert Le Conte has been elected surgeon to succeed the late Dr. John Ashhurst, Jr., whose place he filled as acting surgeon during the latter's illness. Dr. Le Conte is also surgeon to the Children's Hospital, the Bryn Mawr Hospital, and assistant surgeon to the Orthopedic Hospital.

---

OWING to the financial trouble of D. Appleton & Co., the *New York Medical Journal* has been sold, the new owner being Mr. A. R. Elliott, owner and publisher of the *American Druggist*, who conducts an advertising agency in New York. We are glad to note that Dr. Frank P. Foster, its efficient editor, has been retained.

---

SCRIBNER'S MAGAZINE.—The August fiction edition of *Scribner's Magazine* is always a notable one, both for its short stories and the unusual number of illustrations. This year it will be found especially rich in these particulars as well as in other features. The contents include the names of some of the best-known writers and artists of to-day, and, as usual, those of new contributors to the magazine.

---

THE Mississippi Valley Medical Association meets in Asheville, N. C., October 9–11. This excellent association has shown good judgment in meeting at so delightful a place as Asheville, especially during the month of October. It will give opportunity to the physicians of the great Northwest to see what a beautiful country exists in Western North Carolina, and how well adapted it is for climatic treatment of pulmonary troubles. The program already promises to be one of great interest and excellency.

---

DR. A. M. PHELPS, of New York City, president of the Medical Society of the State of New York, attended the annual meeting of the Oregon State Medical Society, held at Portland, June 26 and 27, 1900. Dr. Phelps gave up an attractive trip elsewhere that he had in contemplation, for the purpose of accepting the invitation of the Oregon Society. He read three papers, or demonstrated three subjects, relating to orthopedic surgery. The meeting was the most successful in the history of the society.

---

THE Knights of Honor at their recent convention in Buffalo discussed and decided two very important subjects at their sessions. They considered Christian science and suicide. Christian scientists are admitted to membership and suicides are not barred after five years. The vote of Christian science was 64 to 13. Whether the Knights needed the money or whether Mother Eddy did one of her white mahatma acts from a distance will never be known; but an interesting phase of the question will be the action of a Christian scientist who happens to be appointed on the sick and benefit committee.—*Buffalo Medical Journal.*

---

THE Trenton (N. J.) Board of Health has nailed the tuberculosis flag to the masthead and issued an order placing that disease in the same category with smallpox, diphtheria, scarlet fever and other contagious and infectious diseases. Patients must be isolated and disinfected, and hospitals are not exempt from the provisions of the order. Physicians are required under penalty of fine and imprisonment to report each case within thirty days after diagnosis and to send a sample of the sputum with the report. Trenton is in New Jersey, a State which has stood the jibes of the United States for years, but it has suddenly stepped to the front as a city with a progressive and fearless board of health. Trenton will wipe out tuberculosis, or come very near it with such a rule enforced.

---

CHANGES IN THE FACULTY OF THE UNIVERSITY COLLEGE OF MEDICINE, RICHMOND, VA.—Dr. Hunter McGuire, on account of health, resigned the chair of clinical surgery, and was elected emeritus professor of the same. He was also reelected president of the faculties of the University College. Dr. Hugh M. Taylor was elected professor of practice of surgery and clinical surgery. Dr. Stuart McGuire was elected professor of principles of surgery and clinical surgery. These professors will have different days for their clinical teaching. Dr. Landon B. Edwards resigned the didactic chair of practice of medicine, and was elected to the full chair of clinical medicine. Dr. William S. Gordon was elected professor of practice of medicine, which caused him to resign as professor of physiology. Dr. Stuart H. MacLean, who has been a lecturer in bacteriology, etc., was elected professor of physiology. The title of proctor was done away with, and that of dean substituted, Dr. J. Allison Hodges being elected to fill its duties. The hospital clinical facilities will be greatly improved and enlarged by the addition of some new wards to the Virginia Hospital, which adjoins the University College lot.

---

MR. W. B. SAUNDERS wishes to announce the final accomplishment of a step that he has long had in mind. Feeling that the growth of the business to its present large proportions has been due, not alone to his own exertions, but quite as much to the efficient cooperation of a number of his employees, he has decided to give recognition to such service by associating with himself in business, under the firm name of W. B. Saunders & Company, Mr. F. L. Hopkins, manager of the subscription department, and Mr. T. F. Dagney, manager of the publication department. These gentlemen have been connected with the establishment almost from its inception, and to their capable management of their respective departments Mr. Saunders attributes much of the success that has attended his efforts.

Mr. Saunders believes that this action will strengthen the position of the house in the eyes of the medical profession, as it will secure a permanence of organization that will ensure the perpetuation of the business. Besides this it will obviate the disadvantages incident to a large business that rests entirely upon the shoulders of one person, by permanently attaching to the house those whose ability and experience have contributed in bringing the business to its present state of prosperity. The subscription and publication departments will be conducted as heretofore. The trade book department will be under the management of Mr. W. D. Watson, whose connection with the house has extended over the past eight years, and who has demonstrated his ability to manage that department with efficiency and success.

---

THE twelfth annual meeting of the Tri-State Medical Society will meet at Chattanooga, Thursday, Friday and Saturday, October 11, 12 and 13, 1900, just after the meeting of the Mississippi Valley Medical Association, Asheville, N. C. A rate of one fare for the round trip will be in force on the occasion of the reunion of the Army of the Cumberland and the Spanish-American War Veterans.

### PARTIAL LIST OF PAPERS.

1. The Therapeutic Effect of Iodine Demonstrated by Physiological Action and Pathological Demonstration—L. H. Warner, Brooklyn, N. Y.

2. Simple Inflammation of the Eye—E. H. Jones, Murfreesboro, Tenn.

3. Demonstration of Rectal Valves with Observations on their Pathology—A. B. Cooke, Nashville, Tenn.

4. Continued Malarial Fever—O. E. Williamson, —, Alabama.

5. Rheumatoid and Osteo-Arthritic Conditions of the Feet, Knees and Spine—Michael Hoke, Atlanta, Ga.

6. Xeroderma Pigmentosa—J. R. Gillipsie, Dayton, Tenn.

7. An Epidemic of La Grippe—R. H. Tatum, Chickamauga, Ga.

8. The Use of Serums and Animal Extracts as Medicinal Agents —P. R. Cortelyou, Marietta, Ga.

9. Tolerance, Suggestion and Other Odd Features in Eye Strain—Ross P. Cox, Rome, Ga.

10. The Use and Abuse of Nasal Sprays—Dunbar Roy, Atlanta, Ga.

11. Twenty-two Operations for Mastoiditis—J. M. Crawford, Atlanta, Ga.

12. The Value of Blood Examinations in General Medicine—Claude A. Smith, Atlanta, Ga.

Papers promised from the following, titles not given:

13. J. B. Murfree, Murfreesboro, Tenn.

14. C. G. Savage, Nashville, Tenn.

15. R. E. Fort, Nashville, Tenn.

16. R. F. Glenn, Nashville, Tenn.

17. Bernard Wolff, Atlanta, Ga.

18. J. G. Earnest, Atlanta, Ga.

19. J. B. S. Holmes, Atlanta, Ga.

20. G. H. Noble, Atlanta, Ga.—A Device for the Relief of Bladder Spasm in the Treatment of Cystitis.

21. Wm. P. Nicolson, Atlanta, Ga.

22. Willis F. Westmoreland, Atlanta, Ga.

23. W. S. Elkin, Atlanta, Ga.

24. President's Address—Relation of the Profession to the General Public—R. R. Kime, Atlanta, Ga.

25. Some Anatomic and Histologic points in the Colon and Rectum of Interest to the Surgeon—J. Rawson Pennington, Chicago, Ill.

26. Supra-Pubic Cystotomy for Traumatism with Median Perineal Drainage—J. G. Carpenter, Stanford, Ky.

27. Delirium: Its Differential Diagnosis and Significance—Frank Parsons Norbury, Jacksonville, Ill.

28. Relation of the Mastoid Process to the Cranial Cavity—Geo. D. Keiper, Lafayette, Md.

29. Little Things in the Treatment of Trachoma—L. W. Beardsley, St. Louis, Mo.

30. Title later—S. G. C. Pinkney, Atlanta, Ga.

---

THE American Association of Obstetricians and Gynecologists will hold its thirteenth annual meeting in the assembly room of the Galt House, Louisville, Ky., Tuesday, Wednesday and Thursday, September 18, 19 and 20, 1900, under the presidency of Dr. Rufus Bartlett Hall, of Cincinnati, Ohio.

The following-named papers have been offered:

1. President's Address, R. B. Hall, Cincinnati.

2. Ovarian Fibroma—Case with Microscopical Report—L. H. Laidley, St. Louis.

3. Cholelithiasis—with Report of Cases—H. E. Hayd, Buffalo.

4. Appendicitis during Pregnancy, Charles G. Cumston, Boston.

5. Diagnosis of Ectopic Pregnancy before Rupture, based on Ten Cases, J. F. Baldwin, Columbus.

6. Three Cases of Extrauterine Pregnancy, with Specimens, W. B. Dorsett, St. Louis.

7. The Private Hospital, Joseph Price, Philadelphia.

8. Paper (title undetermined), E. F. Fish, Milwaukee.

9. Paper (title undetermined) C. C. Frederick, Buffalo.

10. Extirpation of the Rectum and Sigmoid per Vaginam, John B. Murphy, Chicago.

11. Paper (title undetermined) H. O. Pantzer, Indianapolis.

12. Paper (title undetermined) J. H. Carstens, Detroit.

13. The Hymen—of What Significance is its Presence or Absence in Determining Virginity, John Milton Duff, Pittsburg.

14. Paper (title undetermined) W. P. Manton, Detroit.

15. Paper (title undetermined) F. Blume, Pittsburg.

16. A Satisfactory Method for Suspension of the Uterus, Robert T. Morris, New York.

17. Paper (title undetermined) H. W. Longyear, Detroit.

18. Some Points Regarding Surgery of the Gall-bladder, A. Vander Veer, Albany.

19. Surgery of the Liver and Bile Ducts, W. G. MacDonald, Albany.

20. Observations Respecting Malignant Disease of Pelvic Organs, Augustus P. Clarke, Cambridge.

21. Paper (title undetermined) M. Rosenwasser, Cleveland.

22. Bilateral Celiotomy and Shortening of the Round Ligaments for Complicated Retroversion of the Uterus, A. Goldspohn, Chicago.

23. Paper (title undetermined) W. B. Chase, New York City.

24. Paper (title undetermined) Charles A. L. Reed, Cincinnati.

25. Round Ligament Ventrosuspension of the Uterus, D. Tod Gilliam, Columbus.

26. Paper (title undetermined) L. S. McMurtry, Louisville.

The titles of papers are announced in the order of their reception. The permanent program will be classified and issued about August 25, after which date no further titles can be added or changes made in the printed program.

A cordial invitation is extended to the medical profession to attend the several scientific sessions of the association.

